# Corrigendum to “Positive Effect of Electro-Acupuncture Treatment on Gut Motility in Constipated Mice Is Related to Rebalancing the Gut Microbiota”

**DOI:** 10.1155/2021/9835654

**Published:** 2021-05-08

**Authors:** Mingmin Xu, Lu Wang, Yu Guo, Wei Zhang, Ying Chen, Ying Li

**Affiliations:** ^1^School of Acupuncture–Moxibustion and Tuina, Chengdu University of Traditional Chinese Medicine, Chengdu 610075, China; ^2^Teaching and Research Section of Acupuncture, School of Traditional Chinese Medicine, Jinan University, Guangzhou 510632, China; ^3^Office of Educational Administration, Chengdu University of Traditional Chinese Medicine, Chengdu 610075, China; ^4^Graduate School, Chengdu University of Traditional Chinese Medicine, Chengdu 610075, China

In the article titled “Positive Effect of Electro-Acupuncture Treatment on Gut Motility in Constipated Mice Is Related to Rebalancing the Gut Microbiota” [1], the authors identified that the incorrect file was used for Figure 2 during the preparation of the manuscript. The correct Figure 2 has been verified by the editorial board and is as follows.

## Figures and Tables

**Figure 1:**
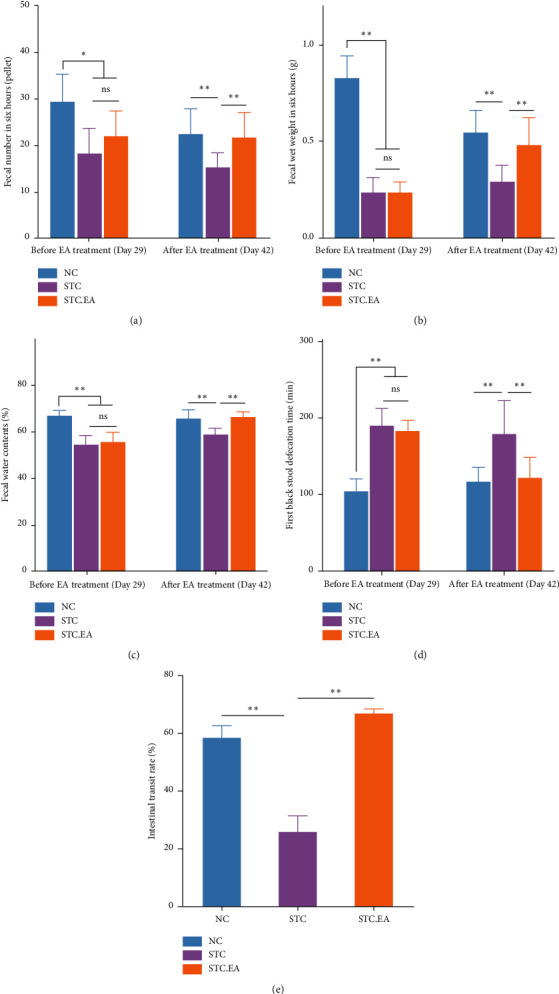
Effects of EA on the defecation status of constipated mice. (a) Fecal number. (b) Fecal wet weight. (c) Fecal water contents. (d) First black stool defecation time. (e) Intestinal transit rate. Fecal parameters (first black stool defecation time and fecal number, weight, and water contents) were measured before (day 29) and after (day 42) EA treatment. Data represent the mean ± SD. ^*∗*^*P*<0.05; ^*∗∗*^*P*<0.01.
